# Naps Do Not Change Delay Discounting Behavior in Young Adults

**DOI:** 10.3389/fpsyg.2018.00921

**Published:** 2018-06-22

**Authors:** Sean O’Connor, Akshata Sonni, Uma Karmarkar, Rebecca M. C. Spencer

**Affiliations:** ^1^Department of Psychological and Brain Sciences, University of Massachusetts, Amherst, MA, United States; ^2^Neuroscience and Behavior, University of Massachusetts, Amherst, MA, United States; ^3^Rady School of Management, University of California, San Diego, San Diego, CA, United States

**Keywords:** discounting, sleep, impulsivity, decision making, naps, economics

## Abstract

When offered a choice of $40 today or $50 later, many would choose the immediate reward over the greater delayed reward. Such behavior is a result of future gains being discounted such that their value is rendered less than that of the immediate gain. Extreme discounting behaviors are associated with impulsivity and addiction. Given recent evidence of sleep’s role in decision making, we tested the hypothesis that sleep would reduce delayed discounting behavior. Twenty young adults (*M* = 20.19 years, *SD* = 0.98 years; 6 males) performed a hypothetical delay discounting task, making a series of choices between an immediate reward (from $0 to $50) or a larger reward ($50) available at a delay of 2, 4, 8, 14, or 22 weeks. Participants performed the task before and after a mid-day nap, and before and after an equivalent interval of wake (within subject, order counterbalanced, wake, and sleep conditions separated by 1 week). As expected, indifference points decreased with longer delays both prior to and following the nap/wake interval. However, the impact of a nap interval on discounting did not differ from the impact of a wake interval. Thus, while sleep has been shown to play an active role in some financial decision-making tasks, a nap is not sufficient to change delay discounting behavior.

## Introduction

The “marshmallow experiment" of Mischel et al. (1989) is known for demonstrating the inability of children to wait for two marshmallows, with them opting instead for a single, immediate marshmallow. While we find humor in these behaviors in children, adults show similar deficits on tasks typically involving monetary rewards. For instance, a person who accepts $50 now over $100 in a year is greatly devaluing the delayed reward relative to the present amount, to the point where the future value is subjectively less than the present value (Mitchell and Wilson, 2012). Delay discounting – choosing an immediate smaller reward over a delayed larger reward – can be economically disadvantageous (Mazur, 1987). Excessive discounting behavior may have its roots in impulsivity (Glimcher et al., 2007), and may underlie other maladaptive behaviors (Bickel et al., 1999; MacKillop et al., 2011; Odum, 2011).

Decision-making behavior can become more impulsive following sleep deprivation. Reynolds and Schiffbauer (2004) observed steeper discounting rates in sleep-deprived individuals compared to those that were allowed to sleep. Sleep deprivation likewise impairs performance on the Iowa Gambling Task (IGT), an affectively guided decision-making task (Harrison and Horne, 2000; Killgore et al., 2006). These deficits from a lack of sleep may reflect reduced function and connectivity of the prefrontal cortex and other brain areas (Drummond et al., 2000; Verweij et al., 2014), causing more impulsive behavior. However, impairments are not consistently observed following sleep deprivation (Acheson et al., 2007; Libedinsky et al., 2013; Demos et al., 2016; Chan, 2017).

While sleep *deprivation* can impair decisions, sleep itself may have positive effects on economic choices with subjective components. For example, people have more positive perceptions toward available choice options following sleep relative to wake (Karmarkar et al., 2017), and affect-guided IGT decisions improve following sleep (Pace-Schott et al., 2012). In addition, amygdala reactivation during REM sleep is thought to contribute to emotion processing, including emotional aspects of decision-making (moral judgements; Cellini et al., 2017).

This raises the question of whether sleep can actively reduce impulsivity during financial choices. To test this, we measured individual discounting rates before (which served as baseline) and after a nap and an equivalent period of wake. A nap paradigm allowed us to assess the active role of sleep compared to a wake control without the confound of sleep depriving subjects. We hypothesized that people would show less discounting after sleep.

## Materials and Methods

### Participants

Participants were 20 young adults 18–30 years (*M* = 20.19 years, *SD* = 0.98 years; 6 males). All participants were screened against high or low sleep quantity (<5 h or >11 h per night), the use of sleep-affecting medications, and the presence of sleep, neurological, or psychiatric disorders.

### Procedure

The protocol was approved by the Institutional Review Board at the University of Massachusetts. All subjects gave written informed consent in accordance with the Declaration of Helsinki.

**FIGURE 1:**
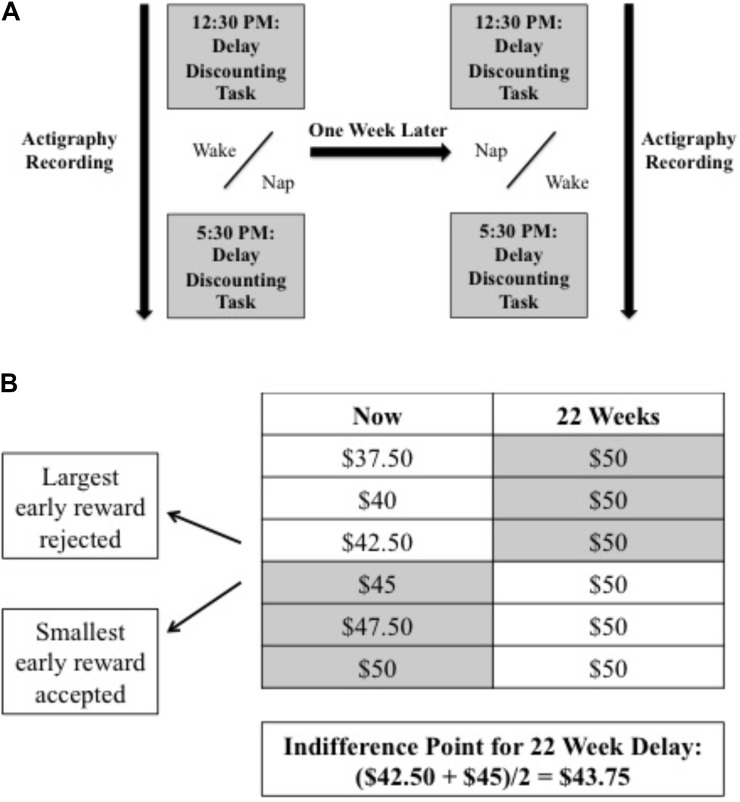
Experimental design indicating **(A)** study procedures (gray bars indicate time points when the delay discounting task was performed) and **(B)** a sample choice (22-week delay) and respective indifference point calculation. Shaded cells represent participant’s choice between the now and later option.

Accordingly, written informed consent was obtained prior to testing. All participants completed a nap and wake condition. In both conditions, participants arrived at the laboratory at 12:30 PM and were instructed to wear an actigraph throughout the day. Participants completed the delay discounting task in the lab and then returned home to either (1) attempt a 3-h nap by turning off the lights and keeping their eyes closed (Nap-First condition) or (2) stay awake (Wake-First condition). They returned to the lab at 5:30 PM to perform the discounting task again (**Figure 1A**). This procedure was repeated exactly 1 week later when participants completed the opposite condition.

### Delay Discounting Task

The task was adapted from Mitchell and Wilson (2012). Monetary choices between a smaller, more immediate reward, or a larger, delayed reward, were presented one pair at a time on a computer screen. Each pair represented a unique combination of two factors: reward magnitude of the immediate option and/or delay of delivery of the larger, later reward. The immediate outcome was always offered at no delay, but with a payout that varied from $0 to $50, increasing in increments of $2.50 (e.g., $0, $2.50). The reward magnitude of the larger, delayed reward was held constant at $50, while its payout delay varied between 2, 4, 8, 14, or 22 weeks. Parametrically varying over these options resulted in a total of 105 monetary choices. All participants were presented with all 105 choices, and their responses were not restricted by a time limit.

### Questionnaires

Habitual sleep quality was assessed using the Pittsburgh Sleep Quality Index (PSQI; Buysse et al., 1989). During each session, current affect (Positive and Negative Affect Scale, PANAS; Watson et al., 1988) and sleepiness (Stanford Sleepiness Scale; Hoddes et al., 1973) were also recorded.

### Actigraphy

The Actiwatch Spectrum was worn on the non-dominant wrist. The actigraph, which uses a triaxial accelerometer to estimate sleep, is deemed valid relative to polysomnography (Mantua et al., 2016). Participants were instructed to press an event marker at “lights off” and at “lights on” for the nap. Data from the watch was scored in 15-s epochs and analyzed using the Actiwatch software. Nap duration and nap efficiency, the amount of time spent asleep relative to time in bed, were calculated (Spencer et al., 2016).

### Analyses

The primary outcome measures were the individual Indifference Points (Mitchell and Wilson, 2012). These were defined as the midway point between the largest immediate reward rejected and the smallest immediate reward accepted for each time delay (2, 4, 8, 14, and 22 weeks; **Figure 1B**). In addition, Discounting Functions (k) were estimated per individual across the six delay points^1^ using the hyperbolic model: V = A/(1+kD), where “V” is the Indifference Point, “A” the delayed reward (set at $50), “D” the length of the delay, and “k” the free parameter that represents the steepness of the discounting (Johnson and Bickel, 2002). Fits were estimated before and after nap and wake using this model with the Matlab *fit* function.

To examine changes in Indifference Points we conducted mixed ANOVAs with within-subjects variables Condition (Wake, Nap), Time Point (before or after nap/wake), and Delay (5 delays), as well as between-subjects factor Order (Wake-First, Nap-First). A similar analysis, (without the Delay factor), was used on the estimated discounting steepness (k). *Post hoc* paired samples *t*-tests were conducted where main effects and interactions were significant.

## Results

All participants stayed awake in the wake condition and successfully napped in the nap condition. Average nap duration was 110.1 min (*SD* = 56.2), with all participants napping for at least 30 min. Naps were efficient, with an average sleep efficiency of 92.05% (*SD* = 5.85). None of the participants reported poor subjective sleep quality (PSQI scores <7; *M* = 3.90, *SD* = 1.74). Negative mood, as assessed with the PANAS, did not differ before [*t*(19) = -0.514, *p* = 0.613] or after [*t*(19) = 0.664, *p* = 0.515] the nap/wake interval. Positive mood was greater before the wake compared to the nap interval [*t*(19) = -2.776, *p* = 0.012], with no difference after the nap/wake intervals [*t*(19) = -0.365, *p* = 0.715].

### Indifference Points

A mixed ANOVA revealed a significant main effect of Delay [*F*(4,72) = 17.651, *p* < 0.001, $\eta_{p}^{2}$ = 0.495], such that Indifference Points were lower for longer delays, consistent with prior studies (e.g., Laibson, 1997). There was a weak main effect of Time Point [*F*(1,18) = 4.003, *p* = 0.061, $\eta_{p}^{2}$ = 0.182] qualified by a significant Time Point x Delay interaction [*F*(4,72) = 2.619, *p* = 0.042, $\eta_{p}^{2}$ = 0.127], such that Indifference Points were higher for long delays following time spent away from the decision task, regardless of sleep.

**FIGURE 2:**
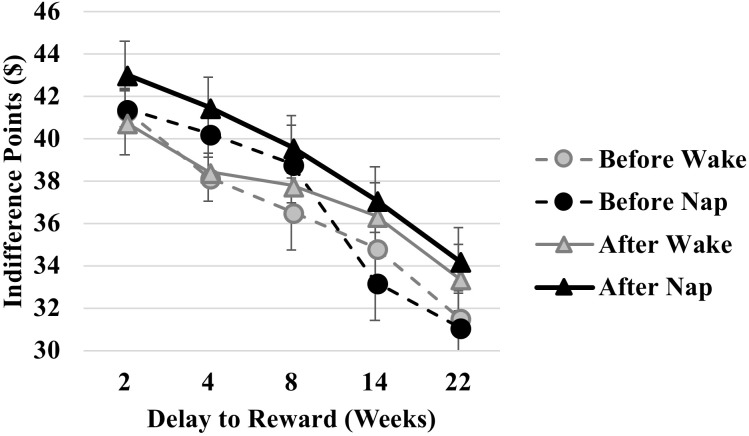
Comparison of indifference points across time delays (2, 4, 8, 14, and 22 weeks) before (circles) and after (triangles) an afternoon nap (black symbols/lines) and equivalent interval awake (gray symbols/lines).

The Condition × Delay interaction was significant [*F*(4,72) = 3.029, *p* = 0.023, $\eta_{p}^{2}$ = 0.144]. *Post hoc* paired samples *t*-tests revealed higher Indifference Points – reflective of less discounting – in the Nap condition compared to the Wake condition for the 4-week delay only [*t*(19) = -2.202, *p* = 0.040, Cohen’s *d* = 0.492; **Figure 2**). However, given that this analysis collapses across Time Point (before/after interval), we presume this to reflect baseline differences in the conditions. Indeed, the Condition × Time Point [*F*(1,18) = 0.607, *p* = 0.446] and Condition × Time Point × Delay [*F*(4,72) = 0.695, *p* = 0.598] interactions were not significant.

While the Condition × Order interaction was significant [*F*(1,18) = 4.599, *p* = 0.046, $\eta_{p}^{2}$ = 0.203], such that Indifference Points were higher for the Nap Condition in the Wake-first group compared to the Nap-first group, the 3-way interaction of Condition × Order × Time Point was not [*F*(4,18) = 0.315, *p* = 0.582, $\eta_{p}^{2}$ = 0.017], ruling out the possibility that any sleep related changes in Indifference Points were related to whether the individuals had completed the task before. No other main effects or interactions were significant (*p* > 0.2).

### Discounting Functions

We examined the differences in participants’ “k” discount parameters before and after periods of nap and wake using a repeated-measures ANOVA. The Order (e.g., nap/wake session order) covariate was not significant (*p* = 0.742). Consistent with the Indifference Points analyses, there was a marginally significant difference between the steepness of the discount rates overall in the nap and wake tasks within subjects [*F*(1,18) = 4.375, *p* = 0.051, $\eta_{p}^{2}$ = 0.196], and similarly a marginally significant decrease in steepness when comparing k before and after a nap or wake interval [*F*(1,18) = 3.677, *p* = 0.071, $\eta_{p}^{2}$ = 0.170]. However, the interaction between these factors did not reach significance [*F*(1,18) = 1.99, *p* = 0.175, $\eta_{p}^{2}$ = 0.100], indicating that the magnitude of the changes was not different with sleep.

## Discussion

In this study, we find that sleep, operationalized as a mid-day nap, does not modulate delay discounting behavior. As expected, indifference points were lower for longer delays. Although there was a significant Delay × Condition interaction for the Indifference Points, the Delay × Condition × Time Point interaction was not significant. Because we randomized order, used a within-subject design, and found the Delay × Condition effect only at the 4 weeks delay, we assume the Delay × Condition interaction to be a false positive.

A similar lack of differences between the effects of nap and wake intervals was observed when fitting a hyperbolic discounting model to the indifference points across the range of delays. This analysis is useful as it offers a summary of the general steepness (k) of an individual’s overall discounting function. However, it should be noted that fitting these functions to a relatively small number of delays (6) is sensitive to variation (and noise) in the data.

These results run counter to our central hypothesis, arising from prior evidence that a bout of sleep can influence some forms of consumer and/or economic decisions (e.g., Pace-Schott et al., 2012; Karmarkar et al., 2017). That earlier research employed an overnight sleep interval, which raises the possibility that a longer sleep bout is necessary to impact delay discounting behavior. It has been suggested that delay discounting may be less sensitive than other tasks of inhibitory control and decision making (Acheson et al., 2007). In our task, the mean nap length was greater than 90 min, suggesting many subjects likely reached REM sleep (see Milner and Cote, 2009), but it may be that late-night REM is needed to see sleep-relevant changes. Others have suggested that a longer interval of sleep deprivation (as opposed to our mid-day napless condition) may be necessary to see such effects (Libedinsky et al., 2013; see also Killgore et al., 2006). Alternatively, this study, combined with other recent findings could point to delay discounting behavior as independent of sleep (e.g., Acheson et al., 2007; Libedinsky et al., 2013; Demos et al., 2016; Chan, 2017). Our results motivate future studies employing longer periods of sleep (one or more overnight sleep bouts) to distinguish between these possibilities.

There are several limitations to consider. First, we report a null result, though we believe it is important to document to avoid publication bias and to move the field forward (Matosin et al., 2014). Second, as noted, our design would have benefited from the use of polysomnography in the nap interval. An in-lab study would also have allowed us to carefully control the nap and wake behaviors. Finally, this study used hypothetical rewards which may not replicate fully incentivized behavior (Paloyelis et al., 2010).

Overall, understanding sleep’s role in these types of behaviors is important in supporting healthy decisions in impulsive populations as well as other meaningful groups. This paper expands the research on sleep and decision-making, and finds that delay discounting is not sensitive to short periods of sleep, compared to non-deprived wake.

## Author Contributions

RS contributed to the study conceptualization. SO, AS, and RS contributed to the study design. SO contributed to the data collection. SO and AS did the data analysis. SO, AS, UK, and RS contributed to the data interpretation and manuscript writing.

## Conflict of Interest Statement

The authors declare that the research was conducted in the absence of any commercial or financial relationships that could be construed as a potential conflict of interest.

## Footnotes

1 Given the sparse number of data points and the standard delayed reward, the canonical data point of zero delay and $50 indifference was explicitly included in the fits.
